# Photocatalytic degradation of indigo carmine dye by hydrothermally synthesized graphene nanodots (GNDs): investigation of kinetics and thermodynamics

**DOI:** 10.1039/d4ra02476a

**Published:** 2024-07-31

**Authors:** Saba Jamil, Rabia Afzal, Shanza Rauf Khan, Mehwish Shabbir, Norah Alhokbany, Songnan Li, Muhammad Ramzan Saeed Ashraf Janjua

**Affiliations:** a Super Light Materials and Nanotechnology Laboratory, Department of Chemistry, University of Agriculture Faisalabad 38000 Pakistan; b Department of Chemistry, College of Science, King Saud University Riyadh 11451 Saudi Arabia; c Harbin Normal University, Songbei Campus Harbin 150026 China; d Department of Chemistry, Government College University Faisalabad Faisalabad 38000 Pakistan janjua@gcuf.edu.pk Dr_Janjua2010@yahoo.com

## Abstract

Graphene nano dots (GNDs) are an intriguing emerging class of materials at the nano scale with distinctive characteristics and exciting potential applications. Graphene oxide was synthesized in a lab setting using a modified version of Hummers' approach and used as a precursor for synthesis of graphene nano dots. Graphene oxide is then treated through hydrothermal treatment to produce GNDs with exact control over their size and form. Synthesized graphene nano dots were subjected to various instruments to study morphology, crystallinity, size and other properties. UV-visible spectroscopy was used to detect the maximum absorbance of light. For functional group identification, FTIR analysis was conducted. X-ray diffraction analysis explained structural composition and various other parameters *i.e.*, crystal size and diameter, which was further verified by Vesta software. Surface morphology of GNDs was analyzed by scanning electron microscopy. AFM analysis of GNDs demonstrates the topography of the surface. The photo degradation of the indigo carmine dye by the GNDs also demonstrates their superiority as UV-visible light driven photo catalysts. To evaluate the results, the thermodynamics and kinetics of the degradation reactions are examined. The effects of several factors, such as temperature, initial concentration, time, pH and catalyst concentration, are also investigated. The data will be analyzed statistically by regression and correlation analysis using dependent and independent variables, regression coefficient and other statistical techniques.

## Abstract 2

All the data have been shared in the manuscript. However, the data are also available on request *via* email from the corresponding author.

## Graphical abstract

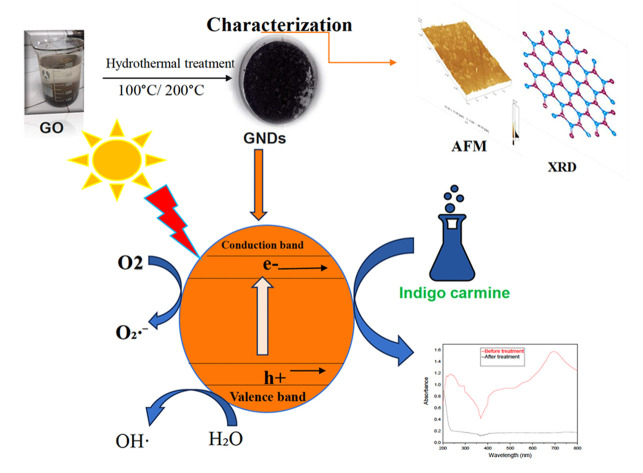

## 1. Introduction

In colloidal science, chemistry, physics, biology and other sciences, nanotechnology is a branch of knowledge that includes the study of phenomena at the nanoscale.^1^ Advances in nanotechnology allowed for the observation and control of material at the molecular and atomic scales, giving rise to the area of *Nano Science*.^2,3^ Materials with diameters typically between 1 and 100 nanometers need to be synthesized, characterized, and manipulated.^4^ Science is extremely interested in graphene, a remarkable two-dimensional nanomaterial made of sp^2^ hybridized carbon atoms arranged in a single layer.^5,6^ Graphene is a material with a high surface area to volume ratio that has a wide range of uses in industry like storing energy, electronics, and sensors.^7–9^ Graphene is positioned as a cutting-edge material with game-changing potential in a variety of technological fields.^10,11^ The extremely high electron velocity even at low energies gives rise to its inherent relativistic nature, which provides an insight into relativistic quantum processes.^12–14^

Graphene oxide is a precursor to graphite made by subjecting it to oxidation. It has functional groups such as carboxyl and hydroxyl bonds, making it soluble in polar solvents such as water.^15–17^ By subjecting graphene to oxidation, functional groups containing oxygen are introduced into the surface of the material, yielding GO.^18^ A member of the graphene family, graphene nanodots, are carbon-based nanoparticles.^19,20^ Graphene nano dots has diameter ranges from few nanometers to tens of nanometers.^21,22^ Graphene nanodots (GNDs) are graphene fragments with precisely controlled dimensions that produce fascinating quantum size effect and exciton confinement.^23–25^ A non-zero bandgap is due to those materials' special size-dependent quantum entanglement effects.^26,27^ The optical and electronic characteristics of GNDs can be tailored due to this controlled band-gap correction.^28^ GNDs have very low toxicity,^29^ high stability,^30^ great electrical conductivity,^31^ and high thermal conductivity.^32^ Because of their exceptional electrical, optical, and mechanical properties, graphene nano dots hold promise for a wide range of applications.^33,34^ Through bandgap alignment, synergistic collaboration, morphological manipulation, light adsorption enhancement, or charge transfer promotion, they can also boost the performance of various catalysts.^35–37^

GNDs can be obtained by a variety of methods, which are generally categorized into two groups: top-down and bottom-up methods.^38–41^ The GND dispersions' size and colloid stability can be adjusted in these circumstances, but the yield is often low and purifying procedures are needed to get the desired result.^42^ To do this, a number of approaches have been suggested, including electrochemical procedures,^43^ hydrothermal/solvothermal methods^16,44^ and oxidative cutting.^45^ In addition, a variety of precursors, including graphite,^46^ graphene oxide, or GO,^47^ carbon fibers^48^ have been employed in these syntheses.^49–51^ Annual global production of synthetic pigments and dyes exceeds 10 000 different varieties.^52^ GNDs are effective nanomaterials for degrading organic pollutant dyes.^53^

A simple and cost-effective bottom up “Hydrothermal method” is used to synthesize GNDs because this process doesn't require much time and intricate equipment's. A modified Hummers' method was used to create GO from recycled graphite, and alcoholic aqueous suspension of GO (without the addition of acids or potent oxidizers) were subjected to hydrothermal treatments using a hydrothermal reactor that allows control over the heating schedule and measurement of the internal pressure produced during the reaction.^54^ Considering the unique properties and possible applications of graphene nano dots, their synthesis is a subject of great scientific interest. Graphene nanodots synthesis is a single step synthesis procedure which reduce the complexity of reaction and increase the efficiency. Hydrothermal method is used which is easily scalable and less cost is required for synthesis process. Our approach of preparation is environmentally friendly, quick, with a high supply of precursors, affordable, and the resulting GNDs have strong biocompatibility, making them more appropriate for environmental applications.

## 2. Materials and methods

### 2.1 Chemicals

Graphite powder, potassium permanganate (KMnO_4_), sodium nitrate (NaNO_3_), sulphuric acid (H_2_SO_4_), ethanol (C_2_H_6_O), hydrochloric acid (HCl) and hydrogen peroxide (H_2_O_2_) were obtained from Sigma-Aldrich. No pretreatment was used before using any of the compounds. For the duration of the trials, distilled water from the lab distillery was used.

### 2.2 Synthesis of graphene nanodots

**Fig. 1:**
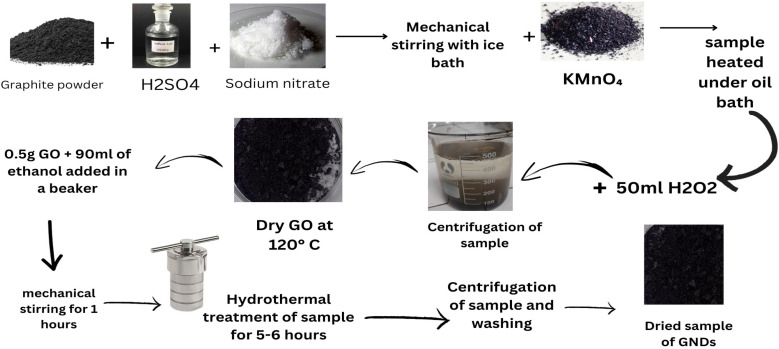
Schematic diagram showing synthesis of graphene nanodots.

The modified Hummers' method was used to synthesize graphene oxide (GO) from powdered graphite. Briefly stated, 23 ml of concentrated sulfuric acid was added while constantly stirring to a mixture of 1 g of graphite powder and 0.5 grams of sodium nitrate. In order to avoid overheating and explosion, the prepared solution was gradually supplemented with 3 g of KMnO_4_ after 1 hour. After 12 hours of stirring at 35 °C, the liquid was further diluted *via* the addition of 500 cubic centimeters of water while vigorously swirling. The suspension was then treated with a 30 percent solution of H_2_O_2_ (5 ml) to guarantee that the reaction with the KMnO_4_ was completed. After filtering, drying, and washing the resultant mixture with HCl and water, respectively, graphene oxide sheets were produced.^55^ The produced GO dense film was then chopped into small flakes after drying, and 100 mg of GO was mixed with 50 ml of 99.8% of the ethanol with 50 milliliters of distilled water to create a solution. This suspension was then subjected to a 40 minutes sonication process to create a homogenous dispersion. The GNDs were created using a hydrothermal method like those previously described.^47,56^ It should be noted, too, that in this research, the solution that underwent the hydrothermal processes had acids or strong oxidizing agents (such as H_2_O_2_) added to it. The alcohol-containing aqueous GO solution was put into a homemade Teflon-coated hydrothermal reactor or thermally treated for two hours at predetermined temperature of 125 and 175 °C. The samples were allowed to spontaneously cool down to the ambient temperature for 12 hours after the reaction was finished. The resulting suspensions were then centrifuged for 3 hours at 4000 rpm. The supernatant was then carefully collected and allowed to dry for 24 hours in a stove at 50 °C. The dried material was then thoroughly mixed by means of an ultrasonic bath using 50 milliliters of distilled water for 20 minutes. To get rid of any remaining large particles or aggregates in the material, the dispersion was twice filtered. Then, the filtrated dispersion was assessed using the techniques listed below. To provide a reference sample for comparison, a control sample was also created by merely adding two milligrams of GO to 100 milliliters of water that had been distilled (*i.e.*, without using any hydrothermal treatment). This suspension was then sonicated for 40 min, and the undispersed material was removed using filtering. This item's label read GO_Ref shown in Fig. 1.^57^

### 2.3 Process for dye degradation based on graphene nanodots

Indigo carmine is a synthetic, water-soluble dye that is frequently used as a colorant in numerous applications. The residue from a textile mill in Faisalabad sold anionic dye (indigo carmine), which is utilized after further purification. In a photocatalytic degradation experiment, the catalytic effectiveness of as-prepared GNDs for the removal of indigo carmine dye in a UV-visible spectrophotometer was investigated. In a measuring flask, 1 g of anionic dyes was dissolved in 1000 ml of distilled water to create the stock solution. From the stock solution, various ppm solutions containing 10 ppm, 20 ppm, 30 ppm, 40 ppm, and 50 ppm were created in order to create the standard curve using UV spectrometer absorption values. A brief explanation of the reaction's steady state dynamics was given. The entire reaction was carried out in a short period of time—120 minutes—and a comparison study of the photocatalytic degradation of dye was conducted to highlight the purpose of basic GNDs in this process.^58^

An aqueous solution of the indigo carmine dye was created and then dispersed by ultrasonication over 10 minutes as part of the GND-based degrading procedure. By pouring either NaOH or a solution of HCl to the desired concentration, the initial pH level of the solution was changed. The degradation rate of indigo carmine was examined using 0.01 ml of GNDs introduced to a quartz cell containing an aqueous solution of the indigo carmine dye as a catalyst. UV-vis measurements were taken at specific time intervals (at *λ*_max_ = 553 nm) to track the change in indigo carmine dye concentration. The formula used to determine the percentage of degradation (*D*%) was *C*_o_ − *C*_*t*_/*C*_o_ × 100, where *C*_o_ and *C*_*t*_ represented the dye's original concentrations at time 0 and time *t*, respectively.

### 2.4 Direct dye degradation mechanism

Another method of photocatalytic degradation of dyes can also take place in the presence of visible light due to their ease of absorption of certain wavelengths of light. This process involves the excitation of dye from its basic state (Dye) to its triplet state of excitation (Dye*) under a visible-light photons (*λ* > 400 nm). A single electron insertion into a conduction band of GNDs further transforms this excited state dye species into the semi-oxidized radial cation (Dye^+^). These trapped electrons react with the dissolved oxygen in the solution to create superoxide radical anions (O_2_^−^˙), which precipitate the creation of hydroxyl radicals (OH˙). The process of oxidation that occurs in organic molecules shown by the equations is mostly caused by these OH˙ radicals.^59^ When the indigo dye undergoes oxidative cleavage intermediary product isatin-5-sulfonic acid is produced. Isatin-5-sulfonic acid undergoes further oxidation and formed carbon dioxide (CO_2_) and water (H_2_O). Following additional breakdown and mineralization, the nitrogen in the dye molecules may finally produce nitrates. Sulfate ions are also formed carbon dioxide (CO_2_) and water (H_2_O) are less harmless then the initial product. These product doesn't contaminate the nutrients and cause less issue for aquatic life as compared to initial substance.

(i) Photo excitation of GNDs:GND + *hν* → GND (e^−^ + h^+^)

(ii) Formation of reactive oxygen species:H_2_O + h^+^ → ˙OH + H^+^O_2_ + e^−^ → O_2_˙^−^O_2_˙^−^ + H^+^ → ˙OH + OH^−^

(iii) Degradation of indigo dye:
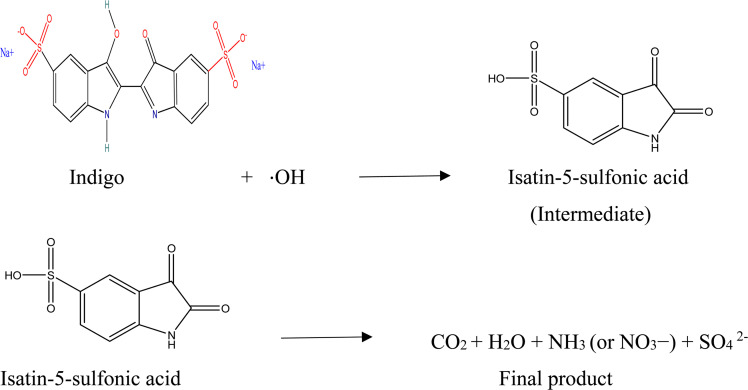


## 3. Results and discussion

### 3.1 UV-visible spectroscopy

**Fig. 2:**
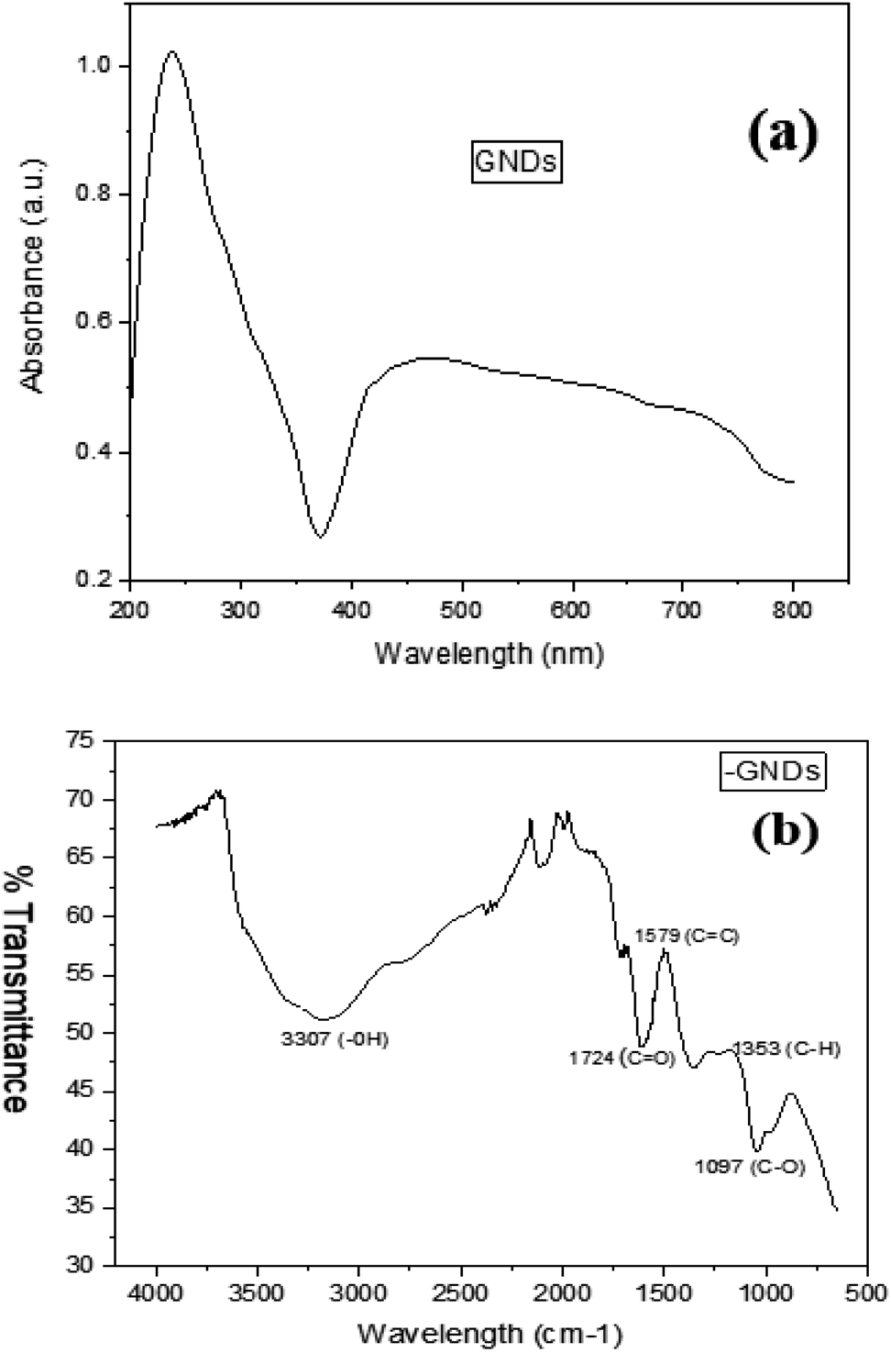
(a) UV-visible (b) FTIR spectra of GNDs.

UV-visible spectroscopy, often known as ultraviolet-visible spectroscopy or just UV-visible spectroscopy, is a technique used to detect the absorption of light. It works within the ultraviolet, UV, or visible (vis) regions of the electromagnetic spectrum. The sample's absorption spectrum, which is produced using UV-vis spectroscopy, displays the range of wavelengths at which light is absorbed by the sample and sheds light on its chemical makeup and structural details. Fig. 2(a) displays the spectrum of UV-vis absorption obtained for the samples that were created; these spectra demonstrate a significant absorption band that is thought to be caused by the absorbed wavelengths of the graphitic structure. UV-vis absorption was used to examine the optical characteristics of GNDs. Fig. 2(a) depicts the spectrum of ultraviolet-visible absorption of GNDs and exhibits a significant tail expanding into the visible range, an aromatic sp^2^ domain-induced π–π* transition absorbance peak around 250 nm and an n–π* transition absorption peak at 360 nm.

### 3.2 FTIR of GNDs

Identifying the functional groups which were placed at the borders of the GNDs is frequently done *via* FTIR analysis. It helps to identify individual groups of functions or chemical bonds, comparing the Fourier transform infrared spectrum for the GNDs to other spectra or databases. Consider the synthesis process, functionalization of the surface, or any other chemical treatments that were performed on the GNDs when interpreting the observed spectrum characteristics. The FTIR spectra of GND are shown in Fig. 2(b). The borders of the GNDs or the basal planes were where functional groups containing oxygen, including carbonyl (1724 cm^−1^), hydroxyl, carboxyl, and epoxy groups, were introduced, according to FTIR research. The broad band between 3200–3500 cm^−1^ indicates that the produced graphene nanodots contain an OH group. Peak at 1353 cm^−1^ can be caused by C–H stretching vibrations, whereas peaks between 1700 and 1800 cm^−1^ are caused by C=O stretching vibrations. These confirm the successful synthesis of graphene nanodots.

### 3.3 AFM analysis

The topography of the surface and topology of graphene nano dots at the nanoscale can be described using a sophisticated imaging method called atomic force microscopy. AFM research has been used to examine the microstructural aspects and particle size distribution of the GND samples.

**Fig. 3:**
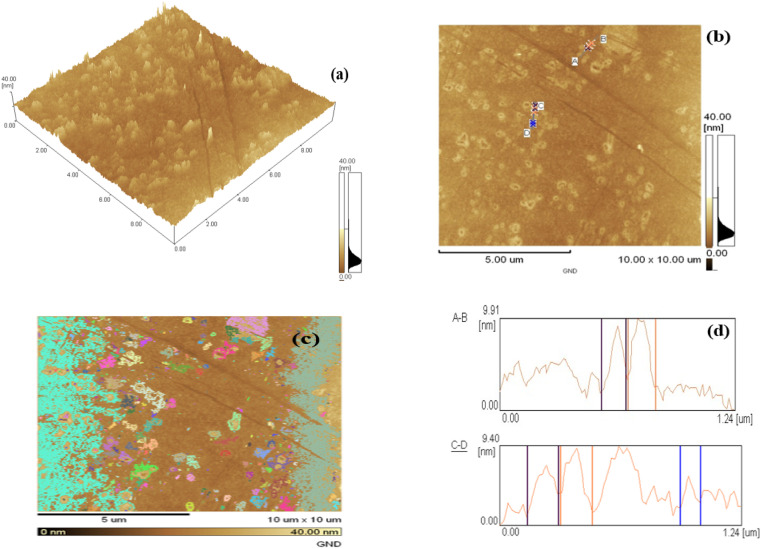
(a) AFM image (b) AFM heat map (c) AFM graph and (d) height profile graph of GNDs.

Fig. 3(a) and (b) displays the GND AFM images. Fig. 3(a)'s depicts that matching AFM image have only one GND layer thin film with nano dots displaying particle sizes that vary from 10–15 nm. 90 percent of the particles were dark brown in color, with sizes between 1 and 10 nm being given to them. AFM analysis indicated that GND diameters were mainly distributed in the range of 20–67 nm, with the average diameter of 40.5 nm. An atomic force microscope's 3D image in Fig. 3(a) and heat map in Fig. 3(b) show that the particles are homogeneous and have the appropriate dispersion. The GNDs' height profile and size were examined using AFM. GNDs are equally spread with few graphene layers, as seen in Fig. 3(c). GNDs have average height of 0.9–1.48 nm as shown in Fig. 3(d). All samples of GNDs have a height distribution between 1 and 10 nm, indicating that most synthetic GNDs are made of few layer (1 to 10 nm) graphene sheets.

### 3.4 XRD analysis

**Fig. 4:**
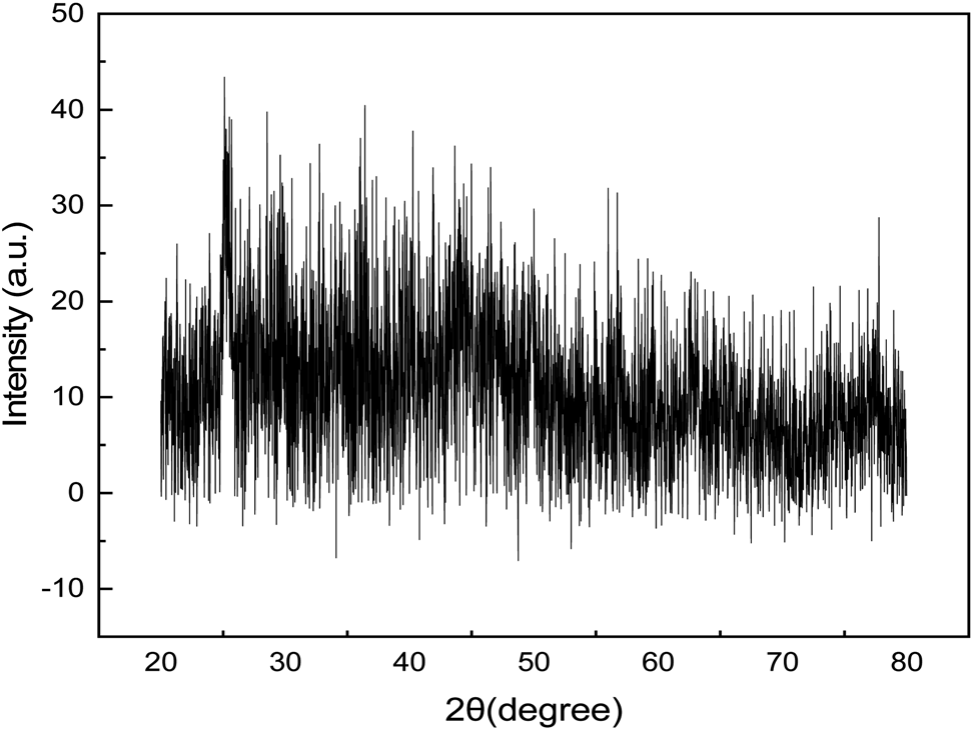
XRD spectra of GNDs.

Fig. 4 displays the synthesized GNDs' XRD pattern. In general, one or more distinct peaks should be visible in the XRD pattern of graphene nanodots. These peaks' locations on the XRD pattern line up with the crystalline lattice planes' interplanar spacing, or *d* spacing. The most noticeable peak for graphene nanodots is frequently seen at 2*θ* of around 25.5°, which matches with the (002) plane of graphitic carbon and carries a *d*-spacing of 0.366 nm. The GNDs' diffraction peak at 2*θ* suggests that the intricate structure of carbon atoms was restored following reduction. The GNDs' larger-spacing value suggests that even after reduction, synthetic GNDs still include functional groups that contain oxygen. The degree of crystallinity and alignment of the graphene nanodots are reflected in the intensity of the XRD peaks. As seen in Fig. 4. Strong, distinct peaks point to a highly crystalline structure. The peak at 2*θ* of 25.5° is narrower, indicating a more consistent and organized structure. The XRD pattern shows other peaks along with (002) peak. These peaks may be indicative of contaminants, secondary materials, or distinct crystallographic phases.

On the basis of the diffraction peak's full width at half of its maximum (FWHM), the mean diameter of the crystal structure of the synthesized GNDs was calculated to be around 2 nm.

**Fig. 5:**
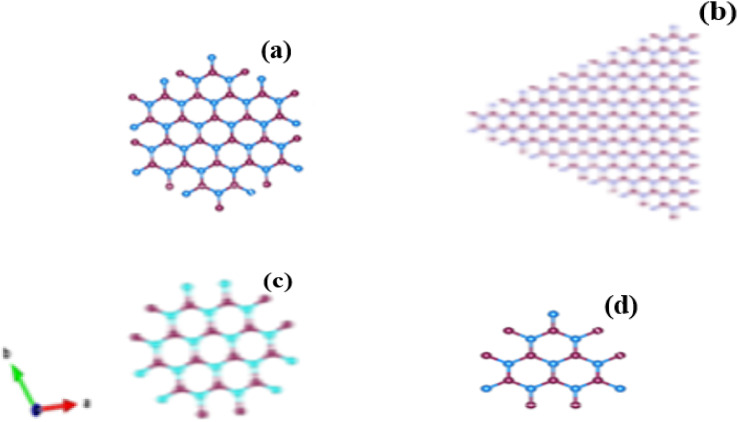
Diagrams representing several graphene nanodot configurations. The shapes that have armchair edges are (a) hexagonal, (b) triangular, (c) hexagonal with zigzag edges, and (d) triangle with zigzag edges.

The Debye–Scherrer formula was used to compute the average diameter,*D* = 0.9*λ*/*β* cos *θ*where *D* is the average diameter of the synthesized GNDs, *λ* wavelength of X-ray, *θ* the Bragg diffraction angle, and *β* full width at half of its maximum (FWHM). Based on the diffraction peak's full width at half of its maximum (FWHM), the mean diameter of the crystal structure of the synthesized GNDs was calculated to be around 2 nm (Table 1 and Fig. 5).

**Table 1:** An overview of the parameters determined using the hydrothermal approach based on the XRD pattern of graphene nano dots

Parameters	Results
Name	Graphene nanodots
Chemical formula	C_57_H_26_O_11_
Crystal system	Hexagonal lattice
Lateral dimensions	<10 nm
2*θ*, *d*-spacing and *hkl*	25.5°, 3.666 Å and (002)
Particle size range	13–23 nm
Diameter	2 nm

### 3.5 SEM analysis

**Fig. 6:**
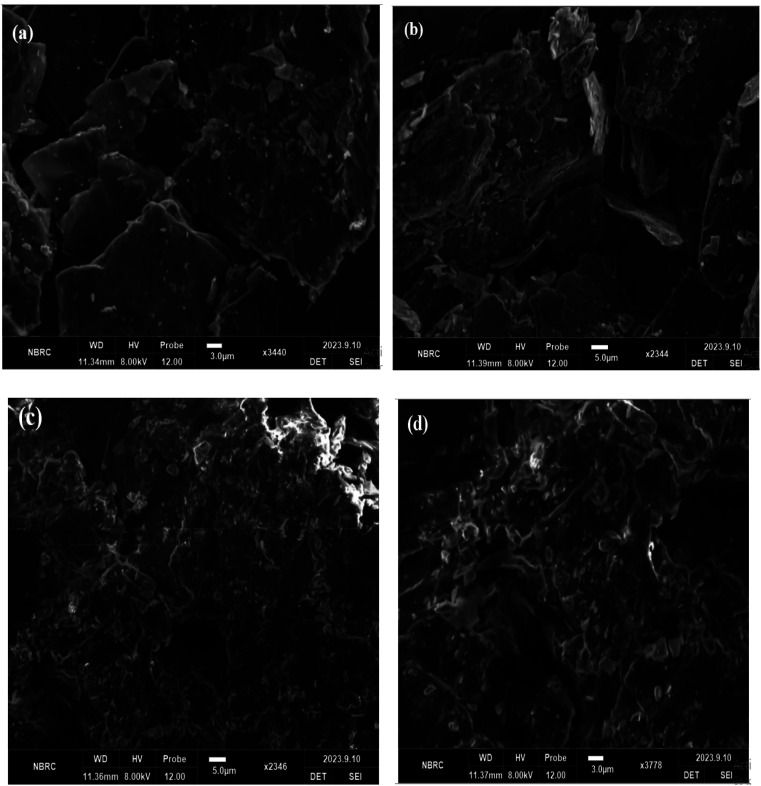
(a and b) Graphene oxide SEM images (c and d) SEM analysis of graphene nanodots.

A potent method for visualizing and describing materials at the nanoscale, such as graphene nanodots, is scanning electron microscopy (SEM). Graphene nanodots are extremely small graphene structures, often only a few nanometers in size. Graphene oxide sheet morphology analysis revealed multilayer graphene's structural makeup. Two-dimensional Graphene oxide's SEM data demonstrate that powder is present. Fig. 6(a and b). Layers of stacked graphene oxide that are somewhat thick are visible. In the SEM images, graphene oxide has a puffy appearance that is caused by numerous oxygen-containing groups on the external layer of graphene oxide, including hydroxyl, carboxylic, epoxy, and carbonyl groups. These groups are found on the outermost layer of graphene oxide because of the product's intense oxidation during the process of synthesizing graphene oxide from graphite.

The SEM pictures of the GNDs at various magnifications, as shown in Fig. 6(c and d), exhibit sphere-shaped morphology. It describes the particle size, which is between 20 and 100 nm. The particle size of graphene oxide is smaller, and the edge aggregates have a close relationship with one another. Less stacks are visible in the graphene oxide pictures (Fig. 6(a and b)), which suggests that the structure on the graphite has exfoliated. In contrast to graphene oxide, graphene nanodots have a rippled surface, smaller, more uniformly sized particles and pores, a randomly ordered aggregate with a thin layer, and are tightly bonded to one another to create an irregular solid as shown in Fig. 6(c and d). The resultant graphene nano dot's structure in Fig. 6(c and d), however, is not just one layer. It is indicated from the SEM graphene nano dots results with 2500 and 5000 times the normal magnification, respectively, which reveals a smoother buildup and a noticeably reduced buildup occur due to exfoliation of structure.

### 3.6 TGA analysis

**Fig. 7:**
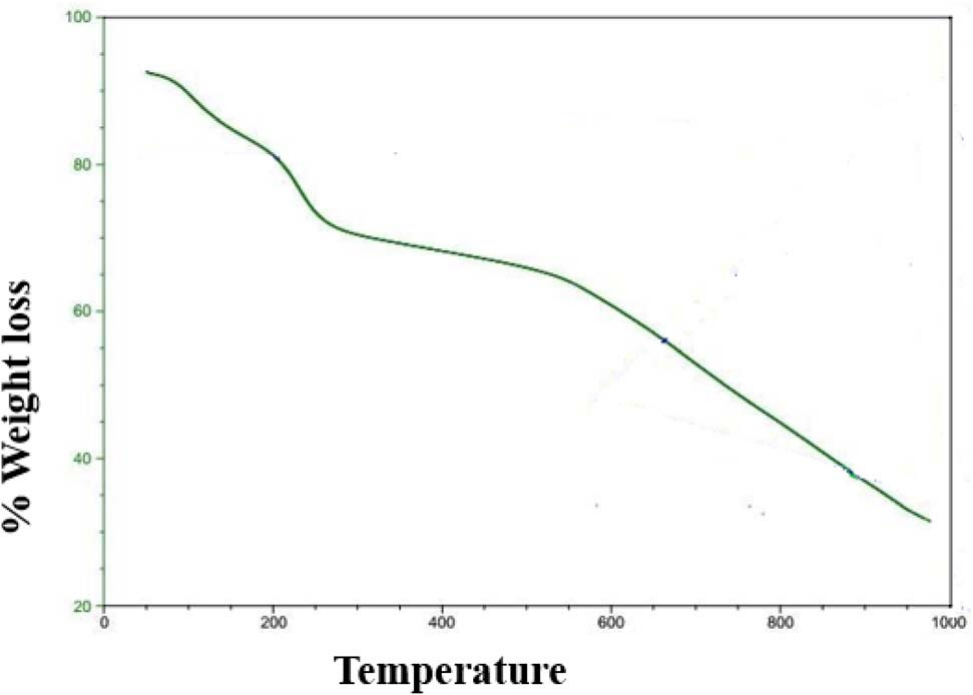
TGA curve of GNDs.

A typical method for examining the heat stability and breakdown behavior of these nanomaterials is the thermogravimetric analysis, or TGA, of graphene nanodots (GNDs). TGA is especially helpful for determining how much weight a sample loses or gains when it undergoes a temperature-controlled ramp in each environment. Using a thermal analyzer (Universal V4.5A TA Instrument) heated at a rate of 10C min^−1^ from 20 to 1000C in Ar, a thermal analysis (TGA) was performed. The thermal stability of the GNDs can be inferred from the point at which they begin to deteriorate or lose weight. A large weight loss was observed in the region 260 to 600 °C, which is caused by the elimination of oxygen-containing groups. The partial reduction of GNDs is what causes fewer oxygen-containing groups of GNDs. The weight fluctuation of GNDs as an indicator of temperature is depicted by the TGA curve. Different functional groups or contaminants may be present as evidenced by the existence of various breakdown stages. Thermogravimetric analysis of GNDs is shown in Fig. 7. Between 150 and 600 °C, the functional groups –COOH present near the edge and –OH groups on the center plane are responsible for most of the weight loss.

### 3.7 Photo-catalytic degradation of indigo carmine using GNDs as a catalyst

In a photo-catalytic degradation experiment, the catalytic effectiveness of synthesized GNDs for the elimination of indigo carmine dye in a setting of UV visible spectrophotometer was investigated. The influence of these variables on the efficiency of removal of the produced nano catalysts was examined using various concentrations of the catalyst dose and hydrogen peroxide (an oxidizing agent), and the amounts were then tuned to provide greater efficacy for the elimination of organic dyes. By conducting the degrading research at 299 to 314 kelvin, the impact of temperature upon the catalytic effectiveness of produced nanodots is evaluated as well. The efficiency of GNDs is clearly visible on graph that catalytic material (GND) synthesized through hydrothermal method is efficient for the degradation of industrial waste water treatment.

### 3.8 Absorption spectra of indigo carmine dye

**Fig. 8:**
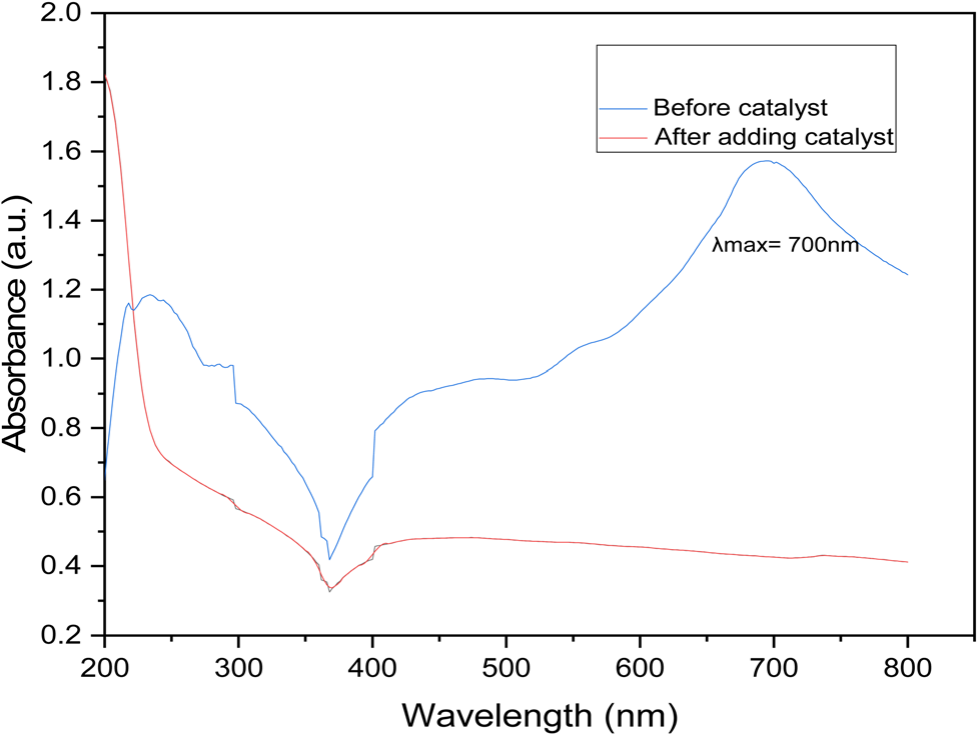
Absorption spectra of GNDS and indigo carmine dye.

It is evident from the UV-vis spectrum that the peaks match the structural features of IC. The IC solution's initial blue hue has a highest signal at 700 nm, which corresponds to the visible range. This is significant to note even though it is evident. The adsorption and following catalytic reactions are usually involved in the degradation of dyes *via* graphene nanodots. Graphene nanodots can adsorb molecules of dye onto their surfaces *via* electrostatic forces and π–π interactions. When the dye solution is treated with GNDs the dye degraded and it absorb at low wavelength depicted in Fig. 8.

The UV absorption standard curve of dye before treatment shows the maximum absorption peak at 700 nm. After degradation the peak goes smoother that indicates that synthesized catalyst is efficient for the degradation of industrial waste water.

### 3.9 Effect of time

**Fig. 9:**
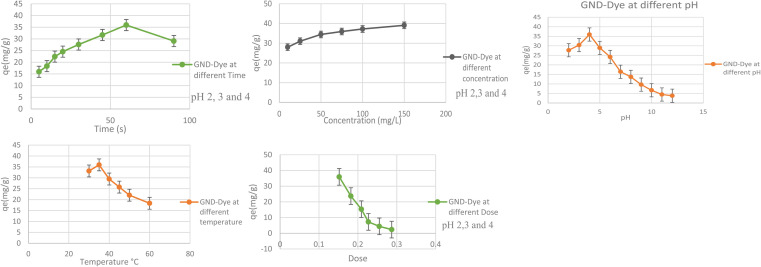
(a) Effect of time. (b) Effect of concentration. (c) Effect of pH. (d) Effect of temperature. (e) Effect of dosage on the use of GNDs to remove dye.

How long GNDs remain in contact with dyes will play a major role in the way they absorb and remove dyes. If the amount of CV dyes taken is plotted against the contact time, we can estimate how long the operation will take. Adsorption is a periodically variable feature that can reveal details about how an adsorption system was designed and put into action. GNDs were used in batch adsorption investigations with contact times varying from 5 to 100 minutes in order to elucidate the impact of duration of contact on both sorption and adsorption procedures.^58^ To determine how to get rid of it, this was done. GNDs were examined for their effects on the dye at pH levels of 2, 3, and 4 with a dosage of 0.05 g, a shaking speed of 110 rpm, a temperature of 35 °C, an amount of 50 mg L^−1^ in a solution, with a contact time parameter that may range from 5 to 90 min. Results are displayed in Fig. 9(a). According to the data displayed below, the absorption of anionic dyes by GNDs started out quickly, slowed down, and eventually achieved equilibrium after 60 minutes. The experiment was conducted for 90 minutes because it was found that the dye adsorption capability peaked at that range of time. When equilibrium was reached, the maximum adsorption capacity was unaffected. After 15 minutes, the sample's adsorption rate starts to decrease and stabilize. As the process progressed through its initial stages, the adsorption rate increased. The maximum adsorption rate was found to be caused by the increased surface area, which provides more opportunities for dye molecule attachment. The degree of adsorption rate decreased with longer contacts times. Adsorption capacity reduced because there were few active binding regions available at saturation because of a decline in surface area. At this point, everything became normal again.

### 3.10 Effect of initial dye concentration

The GND's capacity to adsorb is significantly influenced by the dye solution's initial concentration. This study examined the impact on different concentrations of dye (from 10 to 150 mg L^−1^) for the purpose eradicate indigo carmine pigment from wastewater at the optimal conditions of pH: 2, 3, 4, exposure time of 90 minutes, an at 35 °C, dosage of 0.05 g, with shaking speed of 110 rpm. Results are displayed in Fig. 9(b). According to the results, increasing the dye solution's concentration may accelerate the rate of indigo carmine dye adsorption, as was previously reported. The greatest degree of dye removal was obtained at an amount of dye of 150 mg L^−1^. These pressures help to lower the barrier separating the two phases, allowing the concentration of the dye to reach a stable equilibrium on the adsorbent's surface. As observed in Fig. 9(b) below, as the starting dye concentration rises, more molecules become available to bind to the active regions of the absorbent enhancing the capacity for adsorption while maintaining the same amount of substrate adsorbent. An adsorbent may be able to remove dye more efficiently with larger molecules present, but only up to a point. As the initial concentration of dye rises to 150 ppm, the *q*_e_ value rises. Despite an increase in dye concentration, the ability to absorb has practically reached a plateau at 150 ppm. The active sites of the adsorbents were fully utilized, which is what caused this to occur.

With the higher initial dye concentration, the % clearance decreased. This is because when the initial concentration increased, more dye molecules were present, and the ability of the adsorbent particle got restricted. Additionally, because of the initial concentration increase, the dye molecule's passage from the bulk solution to the surface of the adsorbent is slowed down.

### 3.11 Effect of pH

The effectiveness of the photocatalytic process is significantly influenced by the pH of the solution. Fig. 9(c) shows how the pH affects the indigo carmine dye solution's ability to degrade. GNDs were created, and the impact of pH on their ability to degrade was investigated. The breakdown of indigo carmine was evaluated in a pH range of 2.0–12.0 in the context of a 0.05 g dose, a 50 mg L^−1^ concentration, a 35 °C temperature, at 110 revolutions per minute shaker speed, and a 90 minutes contact time. An aqueous solution of the indigo carmine dye was created and then dispersed by ultrasonication over 10 minutes as part of the GND-based degrading procedure. By pouring either NaOH or a solution of HCl to the desired concentration, the initial pH level of the solution was changed. By increasing the pH of the solution, the *D*% increased to its highest level of >95.0% at pH = 4.0. With a further increase in pH, oxygen-containing functionalities that include carboxylic groups as well as hydroxyl OH are ionized (pH = 4.0), while GNDs at low pH possess undissociated carboxyl (–COOH) groups and epoxy groups. Additionally, the contraction of the double-layer structure brought on by the charge on its surface associated with elevated ionic strengths is what causes the observed drop in *D*% at higher pH levels. As a result, the pH for the subsequent studies was chosen to be between 4.0.

### 3.12 Effect of temperature

The adsorption of dyes may be significantly influenced by temperature, as textile companies usually dump their waste at temperatures that are properly higher. For systems that adsorb waste to function well against actual wastes, temperature is still another crucial component. To eradicate anionic dye from waste from industries water, adsorbents were chosen, and under strictly controlled experimental settings, it was investigated how the temperature range from 30 to 60 °C affected their adsorption ability. Results are displayed in Fig. 9(d). The adsorption capacities of all the tested adsorbent for indigo carmine dye degrading were significantly reduced for the corresponding nanodots, in accordance with the aforementioned findings, and all of the pertinent adsorption processes were categorically exothermic. At 35 °C, all adsorbents produced their best adsorption values. The graph below shows how variations in temperature affect an adsorbent's ability to absorb biological molecules. Each kind of catalysis reaches its peak sorption capacity at 35 °C. An exothermic temperature rise was caused by a physiological chemical reaction. This decrease in adsorption capacity caused the process to produce less heat as the temperature increased. Less adsorption takes place on the exterior of the adsorbent because of the reduced adsorptive interactions among the active sites as well as the molecules of dye or species when the temperature rises.

### 3.13 Effect of catalysts concentration

The dosage, or amount, of an adsorbent determines how effectively it can adsorb chemicals in each situation. Constant working conditions of a pH of 2–4, temperature of 35 °C, dye concentration of 50 mg L^−1^, and speed of 110 rpm, adsorption of dye using adsorbent doses (0.05 to 0.5 g/50 ml), was investigated. In this study, doses between 0.05 and 0.5 g/50 ml being investigated. Results are displayed below when the dosage of the adsorbent is adjusted from 0.05 to 0.5 g while keeping all other parameters constant, the adsorption capacity of GNDs changes. The outcomes show that the highest level of dye removal was achieved with a dose of 0.05 g of GNDs. From 0.05 to 0.5 g, the adsorption capacity fell linearly as adsorbent dosage was raised. The results showed that increasing the adsorbent dosage decreased the amount of indigo dye that was adsorbed, even though the number of unsaturated sites for adsorption increased across the diffusional route length. As the adsorbent dose was increased, the amount of dye in the final solution decreased.

The amount of dye removed initially increased because there were substantial binding sites that allowed dye molecules to adhere, but it then leveled off. In this case, the additional adsorbent was added in an indirect manner to prevent the dye from becoming diluted. Rather, the quantity of surface area that was accessible as well as the existence of functional groups determined how much dye could be removed. To find out, a dye with anionic properties that might stick to the surface due to active site overlap was employed. The adsorbent's capacity was decreased because of this effect at high dosage. The lesser capacity of the dye molecules to absorb at greater adsorbent concentrations is partly due to the fact there simply isn't sufficient adsorbent to adequately cover all of the active sites that are available for binding. This is another element that lowers the amount of ingested solute and contributes to the decline in adsorption capacity.

### 3.14 Kinetics study

The adsorbate absorption is a crucial feature that offers crucial information about reaction pathways and potential uses. Adsorption kinetics measurements are a prerequisite for any batch adsorption system. To select the ideal parameters for a completely batch experiment, understanding of solute absorption kinetics is required. Results on the adsorption phenomena of the Indigo blue dye using various adsorbents have been used to assess the validity of several kinetic approaches, including first-order, second order, and intra-particle diffusion.

### 3.15 Pseudo-first order

The absorption phenomenon may be simplified thanks to the first order. The variation in the concentration of dye over time is directly proportional to P1 according to first-order equations. The pseudo-first-order linear integrated equation is shown below (1898; Lagergren)1
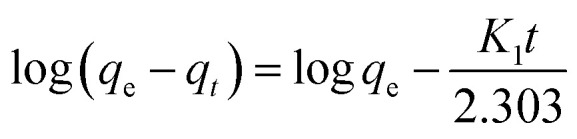
In this case, a pseudo-first-order rate constant, indicated by the number *k*, is used as a substitute. *T* is brief for minute. The line illustrating the relationship between log(*q*_e_ − *q*_*t*_) and *t* was plotted to generate theoretic values for *q*_e_ and *k*_1_; the results were then derived from slope or intercept (Yıldız *et al.*, 2023).^60^ All values are listed in Table 2.

**Table 2:** Kinetic modeling of data for indigo carmine dye adsorption

Kinetic models	Graphene nanodots
**Pseudo first order**	
*k*_1_ (L min^−1^)	−0.0095
*q*_e exp._ (mg g^−1^)	35.93073
*q*_e cal._ (mg g^−1^)	16.51581
*R* ^2^	0.4334

**Pseudo second order**	
*k*_2_ (g mg^−1^ min^−1^)	0.00013
*q*_e exp._ (mg g^−1^)	35.93073
*q*_e cal._ (mg g^−1^)	33.22259
*R* ^2^	0.9709

**Intraparticle diffusion**	
*K*_pi_ (mg g^−1^ min^−1/2^)	1.8113
*C*_i_	12.987
*R* ^2^	0.7372

### 3.16 Pseudo-second order

Effective application of second order allows comprehension of the absorbing process throughout the entire contact period. Second-order differential equation is given.2
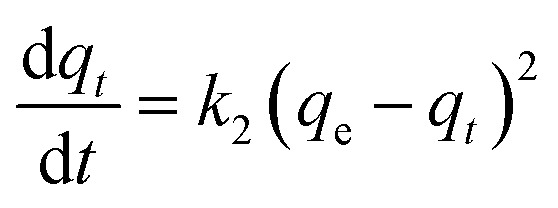
Here, *k*_2_ stands for sorption process' 2nd order rate constant. Following interaction and using boundary conditions second\order linear form is given as3
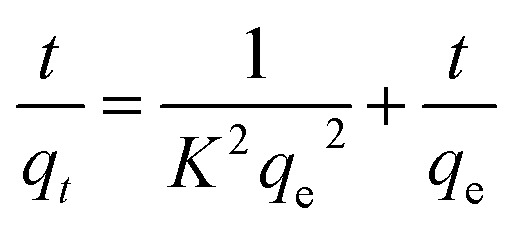


**Fig. 10:**
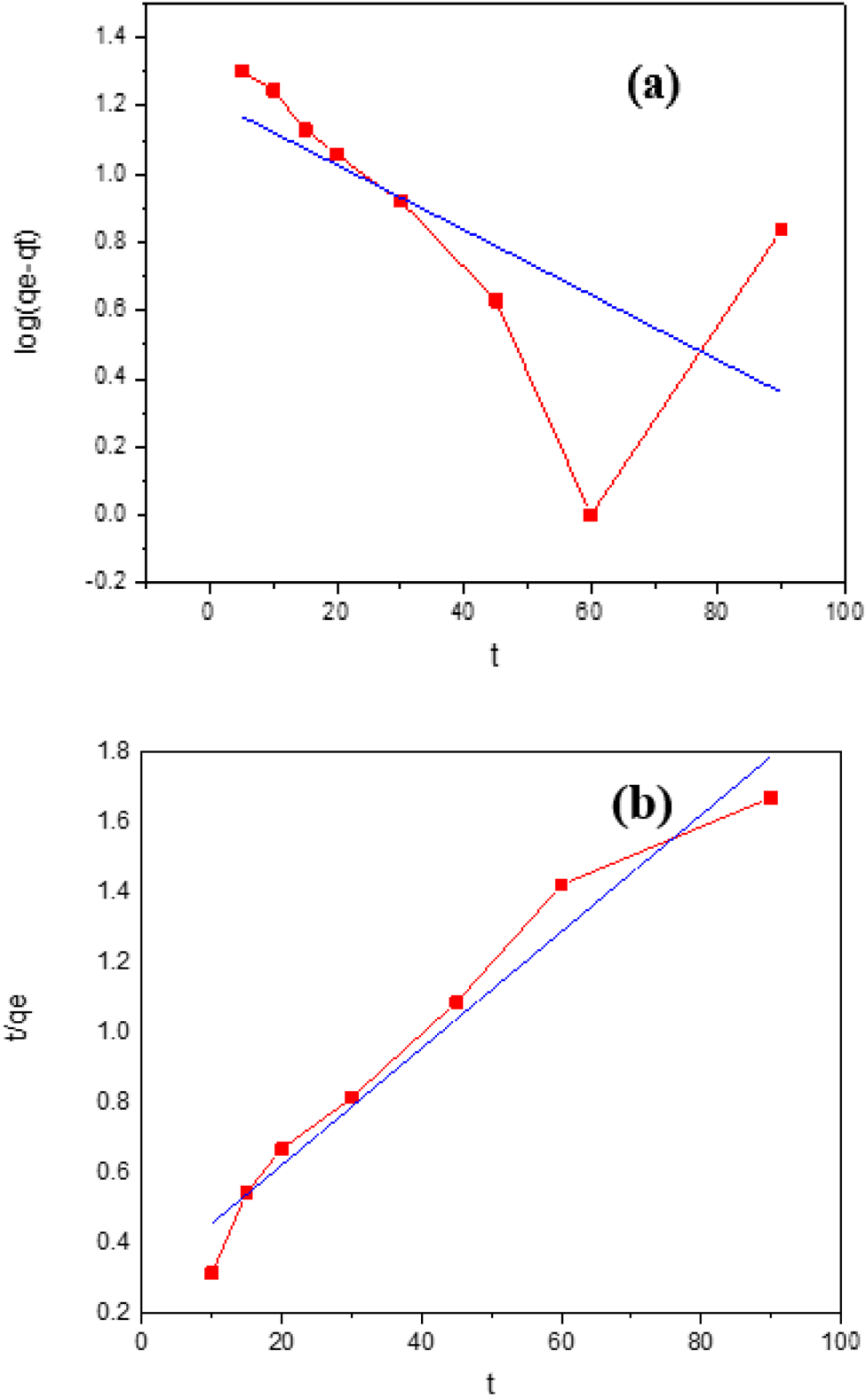
(a) Pseudo first (b) second order kinetic model.

Plotting *t*/*q*_*t*_*vs. t* allows one to determine the hypothesized second-degree rate constant, *k*_2_, in addition to the intercept and slope. Table 2 lists the coefficients of *k*_2_, *q*_e cal._, *q*_e exp._, or *R*^2^ for the absorption of indigo blue using graphene nanodots (Fig. 10).

### 3.17 The kinetic model of intraparticle diffusion

There are numerous processes involved in the absorption of the indigo blue dye molecules through liquid media onto adsorbents. There are three main categories that may be used to categorize the intra particles diffusion processes for the adhesion of the material graphene nanodots on the surface of dye. The first procedure uses molecular diffusion, often referred to as layer or external diffusion, to transfer molecules of adsorbate through the large phase to the dye's outer surface. The second stage of internal diffusion involves the transport of adsorbent molecules from the dye's surface to the inside. The third element is the uptake of absorbent molecules onto the outermost layer of inner holes from the inner area. The slow stage, also known as the rate limiting phase, claims that the capacity of adsorption changes proportionally to *t*^1/2^ rather than the contact period *t*, controlling the speed of the entire adsorption process. The expression of the intra particles diffusion model is as follows:

4 *q*_*t*_ = *K*_pi_*t*^1/2^ + *C*_i_

Here, *K*_pi_ stands for the intraparticle diffusion rate constant, and *C*_i_ stands for thickness.

Graphing the value of the *t* intercept and slope for the *q*_*t*_*vs. t*^1/2^, respectively, will yield their values.

The results of the kinetic adsorption research were utilized to see if different kinetic models could be used. Table 3 contains a list of the kinetic parameters needed for the process of adsorption of Indigo dye on graphene nanodots. As indicated by the correlation coefficient of the results (*R*^2^ = 1), the kinetic data may be explained by a pseudo-2nd-order kinetic model. This result was strengthened by the excellent agreement in the experimental and estimated *q*_e_ values (mg g^−1^). No graphene nanodots adsorption processes notably benefited from using the pseudo-1st order-kinetic model because the expected and actual adsorption capacity values (mg g^−1^) considerably differed. But when applied to the kinetic data, the pseudo-2nd order-kinetic model performed substantially better. Of all the adsorbents employed to remove indigo blue dye, the pseudo-1st order-kinetic model has the lowest correlation coefficient (*R*^2^). In contrast, the pseudo-2nd order kinetics model outperformed both the intraparticle dispersion model and the pseudo-1st order kinetic model.

**Table 3:** Parameters of thermodynamics for IC adsorption on GNDs

Temperature (°C)	Δ*G*° kJ mol^−1^	Δ*H*° kJ mol^−1^	Δ*S*° J mol^−1^ K^−1^
30	1.706407	−41.1676	−128.202

### 3.18 Thermodynamics

The following formulas are used to determine the values of the thermodynamic parameters for the adsorption of IC onto GNDs.5
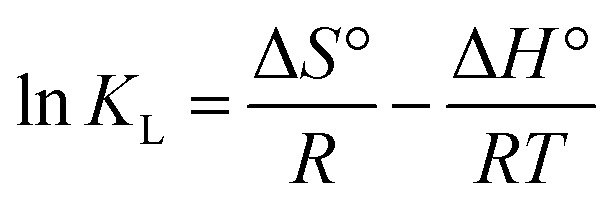

**Fig. 11:**
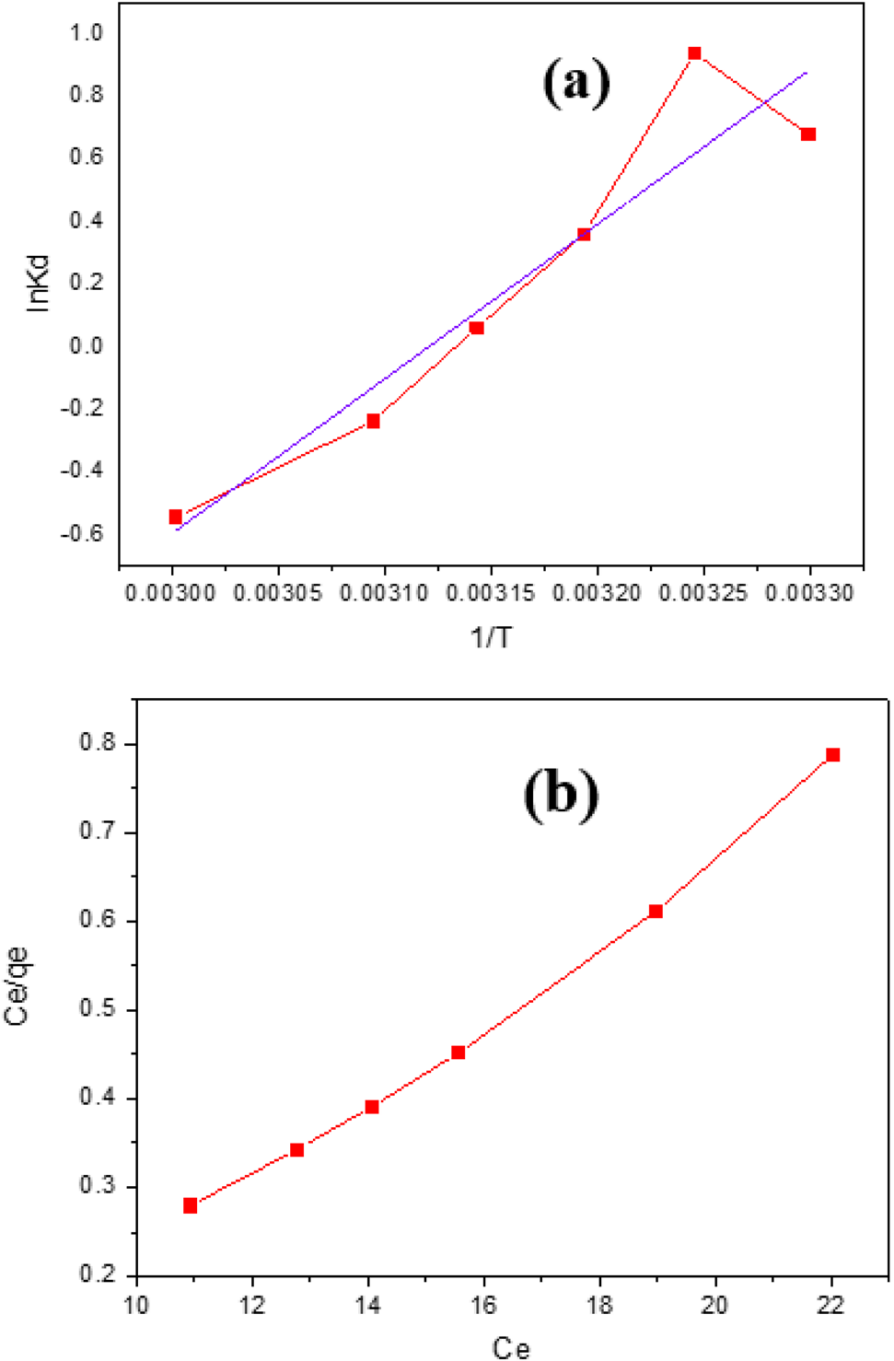
(a) Plot of Van't Hoff (b) Langmuir isothermal plot for adsorption of IC onto GNDs.

6 Δ*G*° = −*RT* ln *K*_L_

where Δ*G*, Δ*H*, and Δ*S* stand for the changes in free energy (kJ mol^−1^), entropy (J mol^−1^ K^−1^), and enthalpy (kJ mol^−1^). The Langmuir constants (L mol^−1^) is denoted by *K*_L_. Fig. 11(a) displays a plot of Van't Hoff, ln *K*_d_*vs.* 1/*T*, and Table 3 lists the values found for the thermodynamic parameters. IB adsorption on GNDs occurs spontaneously in its natural state as the negative Δ*G*. The −ve values for *S*° show that the illness on the solid–liquid border decreased because of every adsorption process for the elimination of anionic dye employing a wide range of distinct kinds of adsorbents. The tendency of *G*° values to be −ve indicates that anionic dye adsorption onto various adsorbents happens spontaneously.

### 3.19 Isotherms

#### 3.19.1 Adsorption isotherms

Understanding adsorption isotherms inside and out is crucial if one is going to be designing an adsorption system because they provide figures that depict the attraction that exists between dye and adsorbent molecules along with data on the adsorption process. Under equilibrium and uniform temperature conditions, the connection between the total amount of adsorbate absorbed by a particular unit of absorbent and the entire quantity present in the sample solution is described by the adsorption isotherm. To verify whether the adsorption system works, researchers must study adsorption isotherms using a variety of isotherm models. Several adsorption isotherms, such as the Langmuir, Freundlich, Temkin, and Harkins–Jura isotherms, can be used to analyze the equilibrium adsorption data from experiments. The study's findings are shown in Table 3.

#### 3.19.2 Langmuir adsorption isotherm

This isotherm's key component, RL, can be measured by using the equation below.RL = 1/1 + *bC*_o_

*C*_o_ stands for the initial dye concentration, RL for the equilibrium parameter, and *b* for the Langmuir constant. The value of RL determines the type of absorption, such as none if RL = 0, unfavorable if RL = 1, linear if RL = 1, or favorable if RL = 1. Using the intercept and slope of the graph of *C*_e_/*q*_e_*vs. C*_e_, the *q*_m_ & *b* may be derived.

#### 3.19.3 Freundlich adsorption isotherm

The Freundlich isotherm of adsorption states that several layers form as adsorption occurs. This demonstrates the interaction between the adsorbed particles, the irregularity of the adsorbent surface, and the uneven distribution of sorption heat (1906; Freundlich). The linear equation for the Freundlich sorption isotherm is shown below.

7 log *q*_e_ = log *K*_F_ + 1/*n* log *C*_e_

In contrast to the adsorption capacity (*q*_e_) & equilibrium dye concentration (*C*_e_), which are stated in (mg g^−1^) and (mg L^−1^), respectively, the Freundlich isotherm's constant (*K*_F_) is given in (mg^1−(1/*n*)^ L^1/^*^n^* g^−1^). The adsorption intensity is given by *n*. The values of the empirical constants that make up the Freundlich isotherm vary on several variables. The extent to which the method of adsorption deviates from the presumption of linearity is determined by comparing the actual and projected values of *n*. In this context, *n* equal to one indicates a linear adsorbent process, *n* below one indicates a chemical adsorption procedure, and *n* over one indicates the potential for applying adsorption technology. It is possible to estimate *K*_F_ and *n* by plotting log *q*_e_*versus* log *C*_e_ in the correct sequence, then determining the slope and intercept of the resulting linear graph. The results of the investigation are displayed in Table 4.

**Table 4:** Equilibrium modeling of data for the removal of indigo carmine dye

Isotherm models	Graphene nano dots
**Langmuir**	
*q*_m cal._ (mg g^−1^)	21.83406
*q*_m exp._ (mg g^−1^)	39.0778
*B*	−90.1117
RL	−983.217
*R* ^2^	0.9912

**Freundlich**	
*q*_m cal._ (mg g^−1^)	28.6169842
*K*_F_	124.9396
*N*	−2.0982
*R* ^2^	0.9777
*q*_m cal._ (mg g^−1^)	21.83406

When utilized using an Indigo blue dye absorbed method, all the investigated adsorbents showed good compatibility for the Freundlich and Langmuir method of adsorption isotherms. This performance demonstrates the diverse composition of the adsorbent particles and implies that dye molecules may be produced on the exterior of the absorbent in both a single layer and several layers. For Freundlich and Langmuir adsorption isotherm, graphene nanodots have *R*^2^ values 0.9912 and 0.9777.

## 4. Conclusion

A simple hydrothermal synthesis technique was successfully used to synthesize graphene nanodots. Graphene oxide was produced by modifying the Offman and Hammer method. At present, the most ecologically friendly technique of producing GNDs is the hydrothermal process that uses graphite oxide (GO) as a starting material. The method that can accomplish mass production is the strong-acidic treatment of graphite. The synthesized product was examined using AFM, SEM, TGA and XRD. Graphene nanodots showed photocatalytic activity by photodegradation of colored indigo carmine dye into colorless compounds and predict how dye degrade when graphene nanodots are added in dye solution and what is the impact of synthesized product on different parameters like time, concentration of dye, pH value when added to dye solution. According to the kinetics analysis, the degradation process adheres to pseudo-first-order kinetics, indicating a direct relationship between the degradation rate and dye concentration. Furthermore, the thermodynamic analysis showed that the degradation of the dye indigo carmine by GNDs is an exothermic, spontaneous process, as shown by the negative values of enthalpy and Gibbs free energy. The impact of various factors on the catalytic performance of synthesized graphene nanodots was also investigated.

## Conflicts of interest

There are no conflicts to declare.
